# Health utility scores of family caregivers for leukemia patients measured by EQ-5D-3L: a cross-sectional survey in China

**DOI:** 10.1186/s12885-018-4855-y

**Published:** 2018-10-03

**Authors:** Hongjuan Yu, Huan Zhang, Jinjin Yang, Chaojie Liu, Chengfang Lu, Hongbin Yang, Weidong Huang, Jin Zhou, Wenqi Fu, Linmei Shi, Yan Yan, Guoxiang Liu, Limin Li

**Affiliations:** 1 The First Affiliated Hospital of Harbin Medical University, Harbin, 150081 China; 2 Affiliate Tumor Hospital of Harbin Medical University, Harbin, 150000 China; 3 School of Health Management, Harbin Medical University, Harbin, 150086 China; 4 School of Psychology and Public Health, La Trobe University, Melbourne, VIC 3086 Australia; 5 Heilongjiang Provincial Hospital, Harbin, 150081 China

**Keywords:** Family caregiver, Leukemia, EQ-5D, Health utility

## Abstract

**Background:**

This study assessed the health related quality of life of family caregivers (FCs) of leukemia patients by using the health utility scores derived from the EuroQol five-dimensional (EQ-5D) questionnaire.

**Methods:**

A cross-sectional survey was undertaken on 306 family caregivers of leukemia patients to assess their health utility using the EQ-5D-3L. Participants were recruited from three hospitals in China’s Heilongjiang province. The health utility scores of the participants were estimated based on the Chinese EQ-5D-3L value set and compared with those of the local general population. Factors predicting the health utility scores were identified through the Kruskal-Wallis analysis of variance and median regression analyses.

**Results:**

FCs had lower health utility scores than the general population (*p* < 0.001). The participants with a lower socioeconomic status had lower utility scores and reported more problems than those with a higher socio-economic status. Better family function and higher social support were associated with higher health utility scores. The type of leukemia, household income, and social support are significant predictors of health utility scores of the FCs. Chronic lymphocytic leukemia, low socio-economic status, and low social support are associated with lower health utility scores of the FCs.

**Conclusions:**

FCs for leukemia patients have lower health utility scores than the local general population, as measured by the EQ-5D-3L. There is an immediate need to address the health concerns of FCs, who play an important role in the Chinese health care system.

**Electronic supplementary material:**

The online version of this article (10.1186/s12885-018-4855-y) contains supplementary material, which is available to authorized users.

## Background

Leukemia is a group of hematologic cancers with malignant clonal proliferation arising from the bone marrow. They may present as an acute condition, such as acute lymphocytic leukemia (ALL) and acute myelogenous leukemia (AML), or as a chronic condition such as chronic lymphocytic leukemia (CLL) and chronic myelogenous leukemia (CML). Leukemia is the most common type of cancer in children. However, most leukemia patients are adults [6, 31].

Overall, the mortality rate of leukemia is very high. In 2012, about 352,000 people were diagnosed with leukemia globally and 75% (265,000) died [36]. In China, the number of leukemia patients ranks at 11 among all cancer cases; but it is the ninth most common cause of death resulting from cancers. In China, it is estimated that the number of new cases of leukemia were about 75,300 and around 53,400 died from leukemia in 2015 [4].

Cancer is a catastrophic event for the family, and can impose serious stress on both the patients and their family members [35]. Over the last few decades, significant progress has been made in the clinical treatment of leukemia in terms of the 5-year survival of the patients. However, the successful treatment of cancer often requires great physical and emotional commitment from family caregivers (FCs). Strong family support is essential for patients who go through the painful process of cancer treatments. The bleak prospect of cancer, coupled with the suffering of patients resulting from cancer treatments, often make FCs feel hopeless, fearful, guilty and regretful. Studies show that FCs caring for cancer patients usually experience worse levels of anxiety, depression, fatigue, sleep problems, and social isolation than FCs who did not care for patients with cancer [17, 28].

In China, a serious shortage in the nursing workforce is prevalent. According to the national statistics, China has 1.8 nurses per thousand population, well below the average (8.8) of Organisation for Economic Co-operation and Development (OECD) countries [23]. This indicates that FCs in China play an even greater role than their OECD counterparts as an important social source and emotional support for cancer patients [3]. In China, family members have a strong feeling of obligation and provide a wide range of care for patients that is otherwise provided by nurses, not only in homes but also in hospitals as well [10, 16, 41]. In addition, the family of cancer patients has to bear a heavy economic burden and they are highly vulnerable to catastrophic health spending despite the universal coverage of health insurance [19].

Health utility is a concept that has been widely adopted for the economic evaluation of the burden of diseases and the cost-effectiveness of interventional activities. A health utility reflects the preference of a population for different health states [12]. Given the significant role of FCs, it is reasonable to expect FCs to be taken into consideration in the economic evaluation of cancers and cancer interventions. However, there is a paucity of literature assessing the health utility of FCs for cancer patients [14]. Some previous studies have focused on the effects of cancer (including leukemia [24, 29, 43]) on various aspects of the health-related quality of life (HRQOL) of FCs [1]. Although these studies reached the conclusion that FCs of leukemia patients have significantly worse psychological, physical, social and environmental well-being than others [24, 29, 43], the absence of baseline utility scores for cancer FCs has jeopardised efforts to comprehensively evaluate the impact of cancers.

This study aimed to fill the literature gap by determining the health utility scores of FCs for patients with leukemia. We chose leukemia in this study for two reasons. First, empirical evidence shows that leukemia has a greater health impact on FCs than many other diseases [29, 46]. Second, it allows us to explore the associations between various patient conditions (eg. children vs adults, acute vs chronic conditions) and the health utility of their FCs.

## Methods

### Study design and data collection

We conducted a cross-sectional survey in Heilongjiang, a province in China with a medium-sized population (38.12 million in 2015) and economic products ($6386 per capita GDP in 2015) [34].

Three cancer centres were selected purposively because they were located in the capital city (Harbin) of Heilongjiang province serving as major referral centres for cancer patients. All of the three centres were affiliated to a tertiary hospital, providing specialist care to leukemia patients across the entire province. The investigators obtained permission from the participating hospitals to conduct the study and asked for a list of admitted leukemia patients over the period of data collection (July 2015 to February 2016). The eligibility of the participating patients for this study was assessed by their doctors and nurses. The participating patients had to have a dedicated primary family caregiver, this being the FC who provided their most of time to care without receiving any financial compensation. Then, 12 trained postgraduate research students were deployed to these centres to conduct face-to-face interviews using a structured questionnaire (Additional file 1). These interviewers did not have a service relationship with the participants. They approached the selected FCs, explained the purpose and protocol of the study, and sought written informed consent from the participants. The participants were encouraged to self-complete the questionnaire unless they requested assistance from the interviewers.

In total, 349 primary FCs were invited and 314 (90%) completed the questionnaire. Five returned questionnaires were excluded from the final analyses due to missing items that are essential for calculating health utility. This resulted in a final sample size of 306 (88% of the invited participants).

### Measurements

#### Dependent variable - health utility

Health utility is a numeric index, with 0 indicating death and 1 representing perfect health. Usually, it is obtained using a generic HRQOL instrument [26]. In this study, we chose the EQ-5D-3L simply because it is the most commonly used instrument [11] and a Chinese population preference value set was recently made available [21].

The EQ-5D-3L contains five items measuring mobility, self-care, usual activities, pain/discomfort, and anxiety/depression. Respondents were asked to rate their current status and experience at three levels: no problems; moderate problems; extreme problems. Each of the combinations (a total of 243) of the five dimensions was given an index score based on a preference weight derived from the general population [21]. In the Chinese value set, the minimal preference weight is − 0.149, indicating a worse than death status, and the maximal preference weight is 1, indicating full health.

#### Independent variables

Health utility can be determined by many factors. In this study, we adjusted the health utility scores by the socio-economic characteristics of the FCs, such as age, gender, educational attainment, marital status, employment, household income, and relationship to patient.

Previous studies [22, 46] demonstrated that the characteristics of patients impose a significant impact on the need for family care and the level of emotional distress of the FCs. Our questionnaire captured the following data in relation to patient characteristics: age, gender, ethnicity, classification of medical insurance, time of diagnosis, and classification of leukemia. These characteristics were associated with how patients respond to their illness and the potential clinical outcomes of cancer treatments [5].

Workloads have been widely accepted as an important factor influencing HRQOL. High workloads can lead to stress, anxiety and depression [7]. In this study, we measured the average daily hours committed by the FCs for caring for the patient while in hospital and the overall annual load (months) of care. We used the Hospital Anxiety and Depression Scale (HADS) to measure the level of anxiety (7 items) and depression (7 items) of the FCs in the prior week. The level of anxiety or depression of FCs caring for leukemia patients was classified as severe (15–21 summed score), moderate (11–14 summed score), mild (8–10 summed score), or normal (0–7 summed score) [47].

Support from the family and community may alleviate the stress levels experienced by the FCs and subsequently improve their HRQOL [5, 18]. We measured the level of social support of FCs with the validated Social Support Rating Scale (SSRS), which resulted in a total score ranging from 66 to 0 [30, 42]. Respondents were divided equally into two groups: ‘high support’ or ‘low support’. We used the family APGAR (adaptation, partnership, growth, affection, and resolve) scale to assess the level of family support of FCs, which resulted in a total score ranging from 10 to 0 [8, 9]. Respondents were categorised into three groups for the purpose of statistical analyses. The summed score was graded as 0–3 (severely dysfunctional), 4–6 (moderately dysfunctional), and 7–10 (highly functional).

### Data analyses

We reported the means and standard deviations (SDs) of the health utility scores of the FCs, as well as the medians and inter quartile ranges (IQs) of these scores. The distribution of the health utility scores measured by the EQ-5D-3L was biased, with 31.0% of respondents reporting the highest possible score of 1.

We compared the utility scores of the FCs with those of the local (Heilongjiang) general population using the Wilcoxon signed-rank test. Such a comparison was made for the following reasons: (1) Population norms were available from a representative sample of the local population in Heilongjiang as part of the fourth National Health Services Survey (NHSS) 2008, involving 15,875 individuals (from 5530 households) in 13 cities and counties [13]. (2) FCs came from this local population. (3) No comparable FCs for other patients were available.

The independent variables that were associated with the health utility of the FCs were identified through the Kruskal-Wallis analysis of variance (*p* < 0.05) and then entered into a multivariate median regression model (all independent variables were coded or transformed into categorical measurements). Ceiling effects are common in HRQOL studies [13, 37], including the EQ-5D-3L [2]. The literature recommends Tobit regression, censored least absolute deviations, and median regression to deal with data of such a censored nature [13, 14, 37], because they have theoretical advantages over the ordinary least squares estimator [13, 25, 38]. When censoring occurs in less than 50% of cases, median regression (robust to censoring, outliers and heteroskedasticity) is equivalent to censored least absolute deviations [25].

The findings of the median regression model were further confirmed by testing the difference in the prevalence of problems (moderate or extreme problems in mobility, self-care, usual activities, pain/discomfort, and anxiety/depression) in the FCs across different categories of the independent variables using chi-square or Fisher’s exact tests.

Data analyses were conducted using SPSS version 22 and STATA version 11, with a *p* value less than 0.05 being deemed as statistically significant.

## Results

### Characteristics of FCs

The FCs for leukemia patients were mostly parents (43.5%) or spouses (37.3%) of the patients. The majority of FCs were married (94.1%) and had a job (77.1%). More than half (54.6%) were women. The FCs had an average experience of 15.5 months (SD = 6.9) of caring for the leukemia patients. More than 97% of FCs had a certain understanding level about the disease. They spent an average of 17.81 h (SD = 7.21) per day caring for the patients in hospitals. On average, the patients had been diagnosed with leukemia for 21 months. AML (53.3%) and ALL (30.4%) were the two major types of leukemia. The respondents had a mean score of 10.81 for anxiety and 8.17 for depression (Table 1).

**Table 1:** Characteristics of patients and family caregivers (*n* = 306)

Characteristics of family caregivers (FCs)	
Gender (% of women)	167 (54.6%)
Age (years, Mean ± SD)	41.20 ± 10. 81
Ethnicity (% of Han)	296 (96.7%)
Duration of caregiving (Month, Mean ± SD)	15.52 ± 6.90
Hours of caregiving per day (Hour, Mean ± SD)	17.83 ± 7.21
Understanding of the disease (*n*, %)	
Incompletely	7 (2.3%)
Partial	190 (62.1%)
Completely	109 (35.6%)
Relationship to patient (*n*, %)	
Spouse	114 (37.3%)
Parent	133 (43.5%)
Child	43 (14.0%)
Other	16 (5.2%)
Level of education (*n*, %)	
No more than primary school	38 (12.4%)
Middle or high school	202 (66.0%)
University	66 (21.6%)
Marital status (*n*, %)	
Married	288 (94.1%)
Other	18 (5.9%)
Employment (*n*, %)	
Employed	236 (77.1%)
Retired	22 (7.2%)
Unemployed	48 (15.7%)
Religious belief (*n*, %)	
No	260 (85.0%)
Yes	46 (15.0%)
Annual household income (Yuan)	
≤40,000	165 (53.9%)
40,001–79,999	131 (42.8%)
≥80,000	10 (3.3%)
Anxiety (Mean ± SD)	10.81 ± 2.32
Depression (Mean ± SD)	8.17 ± 2.23
Social support (Mean ± SD)	37.00 ± 7.91
Family function (APGAR score, Mean ± SD)	6.76 ± 1.82
Characteristics of patients	
Gender	162 (52.9%)
Age	35.65 ± 20.68
Ethnicity	292 (95.4%)
Types of leukemia (*n*, %)	
ALL	93 (30.4%)
AML	163 (53.3%)
CLL	8 (2.6%)
CML	42 (13.7%)
Medical insurance (*n*, %)	
Yes	278 (90.8%)
No	28 (9.2%)
Duration since diagnosis (Month, Mean ± SD)	21.31 ± 18.37

### Health utility scores of FCs

**Fig. 1:**
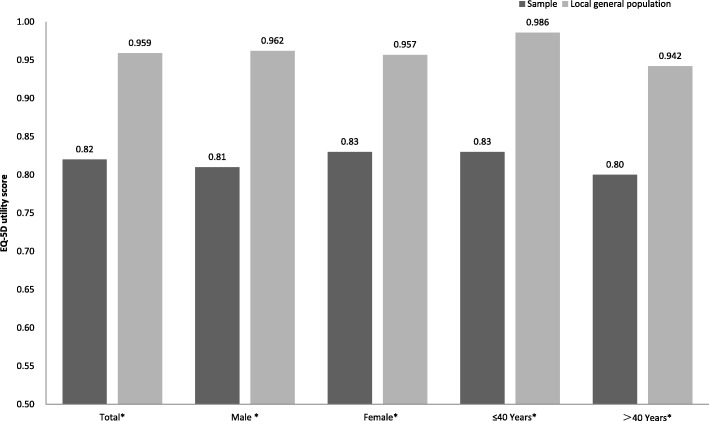
Health utility scores of the FCs for leukemia patients and the local population. * P < 0.001

The FCs who cared for leukemia patients had lower health utility scores than the local (Heilongjiang) general populations (*p* < 0.001, Fig. 1) [13].

The health utility scores of the FCs did not vary across different characteristics of the patients, apart from the type of leukemia (Table 2). The FCs caring for the two chronic conditions had a lower health utility score than those who cared for the two acute conditions.

**Table 2:** Health utility scores of family caregivers

Characteristics	Number	Mean ± SD	Median (range)	*p*
Family caregivers				
Gender				0.546
Male	139	0.81 ± 0.19	0.87 (0.29–1.00)	
Female	167	0.83 ± 0.15	0.87 (0.29–1.00)	
Age (years)				0.092
≤40	154	0.83 ± 0.16	0.87 (0.29–1.00)	
>40	152	0.80 ± 0.17	0.80 (0.33–1.00)	
Relationship to patient				0.010
Spouse	114	0.78 ± 0.19	0.80 (0.29–1.00)	
Parent	133	0.83 ± 0.15	0.87 (0.33–1.00)	
Child	43	0.85 ± 0.17	0.88 (0.29–1.00)	
Other	16	0.91 ± 0.14	1.00 (0.61–1.00)	
Level of education				0.106
No more than primary school	38	0.78 ± 0.16	0.78 (0.51–1.00)	
Middle or high school	202	0.82 ± 0.18	0.87 (0.29–1.00)	
University	66	0.85 ± 0.14	0.87 (0.40–1.00)	
Ethnicity				0.386
Han	296	0.82 ± 0.17	0.87 (0.29–1.00)	
Other	10	0.87 ± 0.12	0.88 (0.71–1.00)	
Religious belief				0.077
No	260	0.81 ± 0.17	0.87 (0.29–1.00)	
Yes	46	0.87 ± 0.14	0.88 (0.51–1.00)	
Marital status				0.724
Married	288	0.82 ± 0.17	0.87 (0.29–1.00)	
Other	18	0.85 ± 0.11	0.87 (0.61–1.00)	
Duration of caregiving (Months)				0.856
≤6	109	0.81 ± 0.19	0.87 (0.29–1.00)	
7–12	69	0.84 ± 0.15	0.87 (0.29–1.00)	
13–24	70	0.81 ± 0.16	0.80 (0.41–1.00)	
>24	58	0.82 ± 0.16	0.87 (0.41–1.00)	
Time spent caregiving per day (Hours)				0.037
0–12	126	0.83 ± 0.19	0.88 (0.29–1.00)	
13–24	180	0.81 ± 0.16	0.80 (0.29–1.00)	
Understanding of the disease				0.606
Lacking	7	0.78 ± 0.16	0.76 (0.53–1.00)	
Partial	190	0.81 ± 0.18	0.83 (0.29–1.00)	
Fully	109	0.83 ± 0.16	0.87 (0.33–1.00)	
Annual household income (Yuan)				0.002
≤40,000	165	0.79 ± 0.17	0.78 (0.29–1.00)	
40,001–79,999	131	0.85 ± 0.17	0.88 (0.29–1.00)	
≥80,000	10	0.83 ± 0.16	0.87 (0.53–1.00)	
Employment				0.021
Employed	236	0.83 ± 0.17	0.87 (0.29–1.00)	
Retired	22	0.71 ± 0.20	0.77 (0.41–1.00)	
Unemployed	48	0.82 ± 0.14	0.79 (0.47–1.00)	
Anxiety (HADS score)				0.063
Normal	23	0.88 ± 0.15	0.88 (0.51–1.00)	
Mild	118	0.83 ± 0.18	0.88 (0.29–1.00)	
Moderate	148	0.80 ± 0.16	0.78 (0.29–1.00)	
Severe	17	0.81 ± 0.17	0.78 (0.51–1.00)	
Depression (HADS score)				0.393
Normal	120	0.82 ± 0.19	0.87 (0.29–1.00)	
Mild	145	0.82 ± 0.15	0.87 (0.29–1.00)	
Moderate	41	0.79 ± 0.16	0.78 (0.33–1.00)	
Social support (SSRSG score)				0.000
Low	153	0.76 ± 0.17	0.78 (0.29–1.00)	
High	153	0.88 ± 0.14	0.88 (0.50–1.00)	
Family function (APGAR score)				0.000
Severely dysfunctional	74	0.75 ± 0.18	0.78 (0.29–1.00)	
Moderate dysfunctional	155	0.82 ± 0.16	0.87 (0.29–1.00)	
Highly functional	77	0.89 ± 0.15	1.00 (0.50–1.00)	
Patients				
Gender				0.381
Male	144	0.81 ± 0.17	0.80 (0.29–1.00)	
Female	162	0.82 ± 0.17	0.87 (0.29–1.00)	
Age(years)				0.303
<15	72	0.81 ± 0.15	0.79 (0.33–1.00)	
≥15	234	0.82 ± 0.18	0.87 (0.29–1.00)	
Ethnicity				0.326
Han	292	0.82 ± 0.17	0.87 (0.29–1.00)	
Other	14	0.86 ± 0.16	0.88 (0.41–1.00)	
Types of leukemia				0.037
All	93	0.83 ± 0.15	0.87 (0.29–1.00)	
AML	163	0.83 ± 0.18	0.87 (0.29–1.00)	
CLL	8	0.66 ± 0.17	0.73 (0.41–1.00)	
CML	42	0.80 ± 0.18	0.78 (0.29–1.00)	
Duration since diagnosis (months)				0.258
0–6	36	0.79 ± 0.19	0.78 (0.29–1.00)	
7–12	82	0.83 ± 0.19	0.88 (0.29–1.00)	
12–24	91	0.81 ± 0.17	0.78 (0.29–1.00)	
>24	97	0.82 ± 0.15	0.87 (0.41–1.00)	
Medical insurance				0.092
No	278	0.77 ± 0.17	0.78 (0.41–1.00)	
Yes	28	0.82 ± 0.17	0.87 (0.29–1.00)	

No significant differences in health utility scores of the FCs were found across gender, age, level of education, ethnicity, marital status, and religious beliefs of the FCs (Table 2). Understanding of the disease, anxiety and depression of the FCs also did not appear as a significant factor associated with the health utility scores of the FCs. Although duration of caregiving was not a significant factor associated with health utility scores of the FCs, those who spent more time daily caring for patients in hospitals had lower health utility scores (*p* = 0.037). The FCs who were a spouse to the patients (*p* = 0.010), currently unemployed (*p* = 0.021), had a low income (*p* = 0.002), low social support (*p* < 0.001), and dysfunctional family (*p* < 0.001) tended to have a lower health utility score than the others.

The median regression model confirmed that the type of leukemia, household income, and social support were significant predictors of health utility scores of the FCs after controlling for differences in other factors. Table 3 presented standardised regression coefficients of these variables, indicating the direction and rate of the change in the utility index as a function of the above variables (both measured in units of their standard deviations). Those FCs who cared for CLL patients had lower health utility scores than those who cared for ALL patients. Higher social support was associated with higher health utility scores. The FCs with a household income in the middle range had higher health utility scores than those in the lower income range. However, with a further increase in income, this difference in health utility scores disappeared.

**Table 3:** Predictors of health utility scores of FCs – results of median regression analyses

Independent variables	Standardised regression coefficient	95% Confidence Interval		*p*
Relationship to patient				
Spouse	−0.076	−0.161	0.009	0.081
Parent	−0.046	−0.132	0.040	0.293
Child	−0.030	−0.123	0.063	0.525
Other (Reference)				
Time spent caregiving per day (Hours)				
0–12 (Reference)				
13–24	− 0.030	− 0.069	0.009	0.133
Annual household income (Yuan)				
≤40,000 (Reference)				
40,001–79,999	0.049	0.010	0.088	0.014
≥80,000	−0.021	−0.126	0.084	0.695
Employment				
Employed (Reference)				
Retired	−0.058	−0.136	0.020	0.143
Unemployed	−0.036	−0.089	0.017	0.184
Social support				
Low (Reference)				
High	0.122	0.078	0.166	0.000
Family function				
Highly functional (Reference)				
Severely dysfunctional	−0.046	−0.110	0.018	0.157
Moderate dysfunctional	−0.016	−0.064	0.032	0.513
Types of leukemia of patients				
ALL (Reference)				
AML	0.000	−0.046	0.046	1.000
CLL	−0.125	−0.239	− 0.011	0.032
CML	−0.035	−0.099	0.029	0.285

### Reported problems of FCs

The reported health problems provided further explanations on the findings revealed by the health utility analyses. Overall, more problems were reported by the FCs in pain/discomfort and anxiety/depression compared with the other domains (Table 4). However, statistical differences in reported problems appeared in different domains for the FCs with different characteristics. The FCs caring for CLL patients were more likely to report problems in mobility and self-care than those who cared for the other types of leukemia patients. Similarly, retired FCs were also more likely to report problems in mobility and self-care than those who were not retired. In contrast, relationship to patients was associated with differences in reported problems in self-care and anxiety/depression (more problems for spouse). Higher intensities of daily care for patients in hospitals and lower household incomes were associated with more reported problems in usual activities, pain/discomfort, and anxiety/depression. Lower social support was the only factor that was associated with more reported problems in all of the five domains (Table 4).

**Table 4:** Reported problems of family caregivers (FCs)

Characteristics	Mobility		Self-care		Usual Activity		Pain/Discomfort		Anxiety/Depression	
	% with problems	p*	% with problems	p*	% with problems	p*	% with problems	p*	% with problems	p*
Family caregiver										
Gender		0.167		0.07		0.769		0.328		0.446
Male	23.74		17.27		17.27		41.73		48.92	
Female	17.37		10.18		18.56		47.31		53.29	
Age(years)		0.040		0.004		0.924		0.498		0.998
≤40	15.58		7.79		18.18		42.86		51.30	
>40	25.00		19.08		17.76		46.71		51.32	
Relationship to patient		0.177		0.035		0.950		0.111		0.04
Spouse	25.44		20.18		18.42		48.25		60.53	
Parent	19.55		9.77		18.05		48.12		48.87	
Child	13.95		11.63		18.60		30.23		41.86	
Other	6.25		0.00		12.50		31.25		31.25	
Level of education		0.308		0.617		0.055		0.016		0.399
No more than primary school	23.68		18.42		31.58		65.79		52.63	
Middle or high school	21.78		12.87		16.83		40.59		53.47	
University	13.64		12.12		13.64		45.45		43.94	
Ethnicity		0.105		0.206		0.314		0.735		0.467
Han	20.95		13.85		17.57		44.59		51.69	
Other	0.00		0.00		30.00		50.00		40.00	
Religious belief		0.356		0.051		0.471		0.141		0.110
No	21.15		15.00		17.31		46.15		53.08	
Yes	15.22		4.35		21.74		36.96		41.3	
Marital status		0.110		0.085		0.882		0.646		0.391
Married	21.18		14.24		18.06		44.44		50.69	
Other	5.56		0.00		16.67		50.00		61.11	
Duration of caregiving (Months)		0.065		0.574		0.734		0.962		0.110
≤6	25.69		11.93		18.35		45.87		51.38	
7–12	11.59		13.04		17.39		42.03		50.72	
13–24	15.71		11.43		21.43		45.71		61.43	
>24	25.86		18.97		13.79		44.83		39.66	
Time spent caregiving per day (Hours)		0.475		0.703		0.044		0.028		0.013
0–12	22.22		14.29		12.70		37.30		42.86	
13–24	18.89		12.78		21.67		50.00		57.22	
Understanding of disease		0.243		0.041		0.243		0.053		0.936
Lacking	42.86		42.86		14.29		0.00		57.14	
Partial	21.05		14.21		15.26		46.32		51.58	
Fully	17.43		10.09		22.94		44.95		50.46	
Annual household income (Yuan)		0.244		0.282		0.026		0.005		0.013
≤40,000	23.03		13.33		23.03		53.33		58.79	
40,001–79,999	16.03		12.21		12.98		35.11		43.51	
≥80,000	30.00		30.00		0.00		30.00		30.00	
Employment		0.022		0.000		0.061		0.216		0.106
Employed	19.92		12.29		16.53		42.8		49.15	
Retired	40.91		40.91		9.09		40.91		72.73	
Unemployed	12.5		6.25		29.17		56.25		52.08	
Anxiety		0.826		0.957		0.577		0.081		0.158
Normal	13.04		13.04		13.04		26.09		34.78	
Mild	20.34		12.71		16.95		40.68		49.15	
Moderate	20.95		13.51		18.24		51.35		56.76	
Severe	23.53		17.65		29.41		41.18		41.18	
Depression		0.176		0.382		0.013		0.469		0.008
Normal	25.00		16.67		11.67		43.33		41.67	
Mild	15.86		11.72		19.31		43.45		54.48	
Moderate	21.95		9.76		31.71		53.66		68.29	
Social support		0.000		0.029		0.011		0.000		0.000
Low	30.72		17.65		23.53		60.78		68.63	
High	9.8		9.15		12.42		28.76		33.99	
Family function		0.023		0.075		0.129		0.000		0.000
Severely dysfunctional	31.08		18.92		24.32		58.11		64.86	
Moderate dysfunctional	18.06		14.19		18.06		47.74		53.55	
Highly functional	14.29		6.49		11.69		25.97		33.77	
Patients										
Gender		0.421		0.566		0.127		0.416		0.840
Male	22.22		14.58		21.53		47.22		50.69	
Female	18.52		12.35		14.81		42.59		51.85	
Age (years)		0.594		0.066		0.154		0.067		0.274
<15	18.06		6.94		23.61		54.17		56.94	
≥15	20.94		15.38		16.24		41.88		49.57	
Ethnicity		0.211		0.482		0.713		0.883		0.920
Han	20.89		13.7		18.15		44.86		51.37	
Other	7.14		7.14		14.29		42.86		50.00	
Types of leukemia		0.008		0.003		0.358		0.227		0.342
ALL	17.2		6.45		13.98		48.39		55.91	
AML	17.79		14.72		19.02		41.1		48.47	
CLL	62.50		50.00		37.50		75.00		75.00	
CML	28.57		16.67		19.05		45.24		47.62	
Duration since diagnosis (months)		0.987		0.902		0.801		0.224		0.641
0–6	22.22		13.89		22.22		52.78		55.56	
7–12	19.51		10.98		19.51		35.37		51.22	
12–24	19.78		14.29		15.38		47.25		54.95	
>24	20.62		14.43		17.53		47.42		46.39	
Medical insurance		0.251		0.467		0.041		0.326		0.517
No	28.57		17.86		32.14		53.57		57.14	
Yes	19.42		12.95		16.55		43.88		50.72	

*p values derived from Chi Square tests

We also found that older FCs were more likely to report problems in mobility and self-care than their younger counterparts. Female FCs reported less problems in self-care than male FCs. The FCs with lower education tended to report more pain/discomfort problems than those with higher levels of education. Those who had a poorer understanding of the disease reported more problems in self-care than those who had a better understanding of the disease. The depressed FCs were more likely to report problems in usual activities and anxiety/depression than those less depressed. Highly functional families were associated with a lower likelihood of the FCs reporting problems in mobility, pain/discomfort, and anxiety/depression.

## Discussion

Family caregivers (FCs) of leukemia patients have lower health utility scores than the local general population. This finding is consistent with other studies which showed that FCs caring for cancer patients, including leukemia patients, had a lower HRQOL [40, 43]. It is evident that the impact of caregiving on the health utility of FCs depends on the type of disease of the patients [14]. The FCs caring for cancer patients are amongst those who are likely to be exposed to the greatest impact. We found that the health utility of FCs varies with the condition of leukemia patients, with CLL having a greater impact on the FCs than other types of leukemia. Unlike in some western countries [15], CLL in China is rare, but has a worse prognosis than other types of leukemia.

Cost-utility analyses have been increasingly used for determining priorities in health care interventions and budgetary decisions. However, little attention has been paid to FCs in such cost-utility analyses [14]. We strongly advocate for the consideration of FCs, not only because FCs are often the primary source of support for patients in many health systems, but also because the poor HRQOL of FCs may impair their ability to care for the patients and eventually result in negative consequences on patient care outcomes. The cost-utility analyses should adopt a value set derived from the local general population. This study shows that the mean health utility scores of the study sample (family caregivers) and the local general population in Heilongjiang are high: 0.82 and 0.959, respectively. This may be a result of relatively younger age structure because the EQ-5D index scores usually decline with age. Previous studies also revealed that Chinese people are less likely to report problems in the EQ-5D compared with most populations in the western countries [39]. In addition, people’s preferences can be quite different under different cultures [33, 39].

The FCs for leukemia patients with a lower socio-economic status have worse health utility than those with a higher socio-economic status. In our study, the lowest health utility of the FCs appeared in those with the lowest household income. They reported more problems in usual activities, pain/discomfort, and anxiety/depression. Similar findings were also reported in other studies [46]. The leukemia patients living in a household with low socio-economic status usually demand more family care because they have limited resources to pay for other supportive services [14, 32, 45]. Sadly, the low health utility of their FCs may jeopardise their capability of caring for the patients. The high demand of family care for the patients with low socio-economic status itself may be blamed for the low health utility of the FCs. We found that higher commitment intensity of care is associated with lower health utility of the FCs, although such an association disappeared after controlling for difference in other factors in the median regression model.

It is important to acknowledge that financial support alone may not be able to offer a solution to the low heath utility problem of the FCs for leukemia patients. We found that the highest income group (≥¥80,000) of FCs had a similar level of health utility as those with the lowest income (≤¥40,000). Health utility scores are derived from HRQOL assessment, which is a subjective measurement. Empirical evidence shows that health utility scores are sensitive to changes in expectations [26]. Often, people’s expectations rise with increased income, which may lower their HRQOL and health utility scores [20].

Social support can play an important role in improving the health utility of FCs for leukemia patients. We found in this study that the FCs with lower social support reported more problems in all of the five domains of EQ-5D-3L, and social support level is a strong predictor of the health utility of the leukemia patients’ FCs in the median regression model. So far, programs designed to support FCs (e.g. respite care) are lacking in China, despite increased appreciation of the contribution of FCs. The decades’ experience of “one child” family planning policy in China has been accompanied with a paradigm shift of supportive services from families to communities [27]. However, it is unrealistic to expect any dramatic decline in the role of family support in health care due to serious shortage in the nursing workforce. FCs will continue to play an essential role in the health care system in China.

This study has the following limitations. First, this study was conducted in three large hospitals in one province, which limits its generalisability. Second, because the participants of this study were recruited in hospitals, the patients they cared for were more likely to be at an advanced stage of cancer [44]. This may bias the estimation of the health utility of the FCs. Third, since this is a cross-sectional survey, no causal inferences can be made.

## Conclusion

FCs for leukemia patients have lower health utility scores than the local general population, as measured by the EQ-5D-3L. The type of leukemia, household income, and social support are significant predictors of health utility scores of the FCs. CLL, low socio-economic status, and low social support are associated with lower health utility scores of the FCs. Cost-utility analyses should consider not only the health utility of patients but also the health utility of FCs. Further studies are warranted to compare the health utility of FCs for different patients.

## Additional file

Additional file 1: Health Related Quality of Life Questionnaire Survey. (DOCX 46 kb)

## Abbreviations

ALL: Acute lymphocytic leukemia
AML: Acute myelogenous leukemia
APGAR: (Adaptation, partnership, growth, affection, and resolve) scale
CLL: Chronic lymphocytic leukemia
CML: Chronic myelogenous leukemia
EQ-5D: EuroQol five-dimensional
FCs: Family caregivers
GDP: Gross domestic product
HADS: Hospital Anxiety and Depression Scale
HRQOL: Health-related quality of life
OECD: Organisation for Economic Co-operation and Development
