# Supplementary material for: Health utility scores of family caregivers for leukemia patients measured by EQ-5D-3L: a cross-sectional survey in China
Source: BMC Cancer. 2018 Oct 3;18:950. doi: 10.1186/s12885-018-4855-y (PMC6171222; doi:10.1186/s12885-018-4855-y)
Supplement: Supplementary file 1 — Health Related Quality of Life Questionnaire Survey. (DOCX 46 kb) [file 12885_2018_4855_MOESM1_ESM.docx]

**Health-related quality of life survey of caregivers in leukemia patients**

Dear Caregiver Friends: (★ The survey is for the patient's primary caregiver, the subject is a family caregiver, and the employment relationship is not included)   Hello! In order to investigate your care pressure and quality of life, meanwhile to reduce your care pressure and improve your quality of life, please complete the following questionnaire. Please fill in according to your actual situation. These answers do not matter "right" and "wrong". Your content will be kept strictly confidential and will not reveal your privacy. Thank you for your cooperation Sincerely!

**1. Situations of caregiver**

1). gender: ( ) 1 male 2 female

2). age: ( ) years old

3). Relationship with the patient who you care for: ( ) ①spouse ②parents ③child ④other

4). Ethnicity: ( )

5). religious beliefs: ( )

6). education level: ( ) ① No more than primary school；②Middle or high school；③University

7). marital status: ①Married ②Unmarried Married ③Divorced ④Widowed

8). Employment: Employed; Retired; Unemployed

9). How long have you been caring for your patients: ( )Months; ( ) hours per day

10). the patient's condition of your understanding: ( ) ①completely；②Partial；⑤Completely

11). the family's income in previous year ( ) ①≤40,000 ②40,001-79,999 ③≥80,000

**2. Conditions of the patient**

12). gender of patient: ( ) ①male；②female

13). Date of diagnosis： ( )year ( )month ( )day

14). age of the patient: ( ) years old

15). name of disease: ( )

16). medical insurance of patient: ( )

17). Ethnicity: ( )

**EQ-5D-3L**

**By placing a tick in one box in each group below, please indicate which statements best describe your own health state today. (√)。**

| **Mobility**  **I have no problems in walking about** ❑  **I have some problems in walking about**  ❑  **I am confined to bed**  ❑  **Self-Care**  **I have no problems with self-care**  ❑  **I have some problems washing or dressing myself** ❑  **I am unable to wash or dress myself**  ❑  **Usual Activities (e.g. work, study, housework, family or leisure activities)**  **I have no problems with performing my usual activities** ❑  **I have some problems with performing my usual activities** ❑  **I am unable to perform my usual activities** ❑  **Pain/Discomfort**  **I have no pain or discomfort**  ❑  **I have moderate pain or discomfort**  ❑  **I have extreme pain or discomfort**  ❑  **Anxiety/Depression**  **I am not anxious or depressed**  ❑  **I am moderately anxious or depressed** ❑  **I am extremely anxious or depressed** ❑ |
| --- |

To help people say how good or bad a health state is, we have drawn a


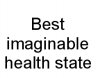
scale (rather like a thermometer) on which the best state you can

imagine is marked 100 and the worst state you can imagine is marked

0.


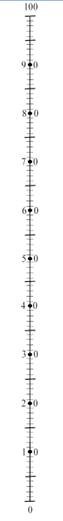
We would like you to indicate on this scale how good or bad your own

health is today, in your opinion. Please do this by drawing a line from

the box below to whichever point on the scale indicates how good or

bad your health state is today.

| Your own  health state  today |
| --- |


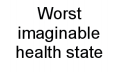


Please read each of the following items and draw a circle on the emotional score that best matches your past month. Don't think too much about the answers to these questions, and the answers you make immediately are often more in line with the actual situation.

|  | Yes  definitely | Yes  sometimes | No, not much | No, not  at all |
| --- | --- | --- | --- | --- |
| 1 .I wake early and then sleep badly for the rest of the night. |  |  |  |  |
| 2. I get very frightened or have panic feelings for apparently no reason at all. |  |  |  |  |
| 3.I feel miserable and sad |  |  |  |  |
| 4.I feel anxious when I go out of the house on my own. |  |  |  |  |
| 5. I have lost interest in things |  |  |  |  |
| 6. I get palpitations, or sensations of `butterflies' in my stomach or chest. |  |  |  |  |
| 7. I have a good appetite. |  |  |  |  |
| 8. I feel scared or frightened. |  |  |  |  |
| 9. I feel life is not worth living. |  |  |  |  |
| 10. I still enjoy the things I used to. |  |  |  |  |
| 11. I am restless and can't keep still. |  |  |  |  |
| 12. I am more irritable than usual. |  |  |  |  |
| 13. I feel as if I have slowed down |  |  |  |  |
| 14. Worrying thoughts constantly go through my mind. |  |  |  |  |

The purpose of the following question is to understand the satisfaction level of your family function, please answer it truthfully, thank you!

|  | Almost Always | Some of the Time | Hardly Ever |
| --- | --- | --- | --- |
| 1.I am satisfied that I can turn to my family for help when something is troubling me. |  |  |  |
| 2.I am satisfied with the way my family talks over things with me and shares problems with me. |  |  |  |
| 3. I am satisfied that my family accepts and supports my wishes to take  on new activities or directions. |  |  |  |
| 4.I am satisfied with the way my family expresses affection and  responds to my emotions, such as anger, sorrow, and love. |  |  |  |
| 5.I am satisfied with the way my family and I share time together. |  |  |  |

The following questions are used to reflect your support from society. Please answer the questions according to your actual situation. Thank you for your cooperation.

   1). How many close friends do you have to get support and help?( ) (Select one)

    (1) No one (2) 1-2

    (3) 3-5 (4) 6 or more

2). In the past year, you have: ( ) (only choose one)

    (1) Stay away from family and live alone.

    (2) The residence changes frequently and most of the time lives with strangers.

    (3) Live with classmates, colleagues or friends.

    (4) Living with a family member.

3). You and your neighbors: ( ) (select only one)

    (1) Never care about each other, just nod.

    (2) It may be a little concerned if you encounter difficulties.

    (3) Some neighbors are very concerned about you.

    (4) Most neighbors are very concerned about you.

4). You and colleagues: ( ) (select only one)

    (1) Never care about each other, just nod.

    (2) It may be a little concerned if you encounter difficulties.

    (3) Some colleagues are very concerned about you.

    (4) Most colleagues are very concerned about you.

5). Support and care from family members ("√" in the appropriate box)

|  | None | few | general | fully support |
| --- | --- | --- | --- | --- |
| A. Couple (lover) |  |  |  |  |
| B. Parent |  |  |  |  |
| C. Children |  |  |  |  |
| D. Brother and sister |  |  |  |  |
| E. Other members (such as scorpions) |  |  |  |  |

6). In the past, when you were in a difficult situation, the sources of financial support and assistance to solve practical problems were:

   (1) There is no source.

   (2) The following sources: ( ) (you can choose multiple options)

A. Spouse; B. Other family members; C. Friend D. Relatives; E. Colleague; F. employer;

G. Official or semi-official organizations such as party and group trade unions; H. Non-official organizations such as religions and social groups; I. Other (please list)_______________________

7). In the past, when you were in a difficult situation, the sources of comfort and concern you have received were:

   (1) There is no source.

   (2) The following sources ( ) (you can choose multiple options)

A. Spouse; B. Other family members; C. Friend D. Relatives; E. Colleague F. employer;

G. Official or semi-official organizations such as the Party and the Communist Party Non-official organizations such as religions and social groups;

I. Other (please list)__________________________________________________________

8). The way you talk when you are troubled: ( ) (select only one)

   (1) Never tell anyone.

   (2) Only 1-2 people who have a very close relationship are told.

   (3) If a friend asks you, he will say it.

   (4) Proactively telling your own troubles to gain support and understanding.

9). How to get help when you are troubled: ( ) (select only one)

   (1) Only rely on yourself and do not accept help from others.

   (2) Rarely ask for help from others.

   (3) Sometimes ask someone for help.

   (4) When you have difficulties, you often ask for help from family, friends, and organizations.

10). For groups (such as party organizations, religious organizations, trade unions, student unions, etc.) to organize activities, you: ( ) (only choose one)

   (1) Never participate

   (2) Occasionally participate

   (3) Regular participation

   (4) Active participation and active activities.
